# Integrated vector management with additional pre-transmission season thermal fogging is associated with a reduction in dengue incidence in Makassar, Indonesia: Results of an 8-year observational study

**DOI:** 10.1371/journal.pntd.0007606

**Published:** 2019-08-05

**Authors:** Isra Wahid, Hasanuddin Ishak, Abdul Hafid, Muhammad Fajri, Sukmawati Sidjal, Armin Nurdin, Naisyah Tun Azikin, Rusdyah Sudirman, Hajar Hasan, Muhammad Yusuf, Imam Bachtiar, William A. Hawley, Ronald Rosenberg, Neil F. Lobo

**Affiliations:** 1 Faculty of Medicine, Universitas Hasanuddin, Makassar, Indonesia; 2 Faculty of Public Health, Universitas Hasanuddin, Makassar, Indonesia; 3 Ministry of Health, Makassar, Indonesia; 4 Centers for Disease Control and Prevention, Atlanta, GA, United States of America; 5 Unicef, Jakarta, Indonesia; 6 Centers for Disease Control and Prevention, Fort Collins, CO, United States of America; 7 Eck Institute for Global Health, University of Notre Dame, Notre Dame, Indiana, United States of America; Centers for Disease Control and Prevention, UNITED STATES

## Abstract

Dengue virus transmission is endemic in Makassar, Indonesia, with the majority of cases reported soon after the start of the annual rainy season. Before 2006, larval source reduction, larvaciding, and reactive routine, outdoor, insecticide fogging campaigns did not result in a reduction in seasonal dengue incidence. Beginning in 2006, village volunteers conducted comprehensive surveys for immature *Aedes* during the dry season, when vector populations were at their lowest. Based on this pre-season vector data, a single additional pre-emptive outdoor fogging with Malathion was conducted once annually before the rains began in villages with a pre-defined proportion of sampled houses positive for *Aedes* immatures. This additional procedure was associated with reduced temporal larval indices as well as an 83% reduction in reported cases during the transmission season over the 8-year period of implementation. Two cities adjacent to Makassar experienced substantial but smaller reductions in dengue incidence; while other cities further from the intervention area did not. This represents the first time an integrated intervention strategy has been coupled with substantially reduced dengue transmission in Indonesia.

## Author summary

Dengue is the most common mosquito-borne viral disease in the world, infecting as many as 400 million people per year. While comprehensive, rigorous vector control can reduce transmission, in practice inadequate coverage, improper application, insecticide resistance, high labor costs, and community indifference hinder its wide implementation and sustainability. Maximum impact in reducing vector populations may be achieved when control interventions are implemented with high coverage and are integrated with other control strategies. A single thermal fogging application will not result in long lasting protection and will have low probability of affecting populations of the *Aedes* vectors and hence disease. This study catalogues an 83% reduction in dengue incidence in the city of Makassar, Indonesia, over an 8-year period, associated with the additional single evidence-based and targeted pre-emptive chemical intervention in *addition* to good case surveillance and an integrated vector management system—attributable to governmental commitment and investment in control of vector-borne disease. A vital part of the observations presented is that they are based on the normal function, capability, and capacity of a Ministry of Health team implementing a community-wide intervention.

## Introduction

Dengue is the most common vector-borne viral disease in the world, infecting as many as 400 million people per year [1]. The disease is caused by four serotypes [dengue-1, -2, -3 and -4] transmitted by *Aedes* mosquitoes [2–5]. *Aedes aegypti* and *Ae*. *albopictus* differ in both their morphology and behaviors–*Ae*. *aegypti* is commonly found indoors, as a container breeder, and in the center of Makassar city; while *Ae*. *albopictus* is found more outdoors, and is prevalent at the edge of the city. A tetravalent vaccine against the four dengue viruses has been licensed commercially but is not yet widely used; while other vaccines are undergoing clinical trials [6, 7]. Safety concerns about the dengue vaccine have also impacted its use. A study found that after being infected with dengue, the vaccine increased the risk of hospitalization by about 50 percent for children not previously exposed to the virus [8]. Vector control remains the most effective method for reduction of dengue, and will likely remain a component of dengue suppression even should a vaccine meet widespread acceptance [9–11]. Transmission occurs in diverse ecological zones and conditions, with key drivers of transmission varying considerably along with these settings [12–17].

While comprehensive, rigorous vector control can reduce transmission, in practice inadequate coverage, improper application, insecticide resistance, high labor costs, and community indifference hinder its implementation and sustainability. Evaluations of vector control tools regularly measure entomological outcomes, rather than disease incidence. The relation between vector density, especially of the mosquito immature stages, and pathogen transmission is complex and difficult to predict [12, 15]. Some studies have demonstrated a limited impact of vector control interventions on entomological indices [9, 18–21]. In other studies substantial reduction of vector indices have had little impact on human disease [11, 22–25]. A recent review [9] concluded that there is a dearth of high quality, standardized studies and reliable evidence demonstrating the effectiveness of dengue vector control methods.

Dengue was first recorded in Makassar, on the southwestern coast of the island of Sulawesi, Indonesia, in 1976. Dengue incidence increased with urban population growth, with about 1,000 cases reported annually to the Ministry of Health (MoH) by 2003. Reported cases tended to represent more serious illness and likely were a fraction of true incidence [4, 26–29]. In response to the increase in dengue cases, Makassar initiated an integrated vector campaign consisting of three initiatives starting in the 1980s. First, a household-conducted larval habitat elimination campaign called 3M (Menutup–to cover; Menguras–to empty; and Mengubur–to bury) was initiated. Second, both local community members and MoH staff conducted larvaciding using Abate (temephos). Abate was made available at local clinics for the community to use in water containers with larvae. Community training was routinely conducted on the identification of immature habitats, *Aedes* larvae, and the use of larvacide, with mosquito control regularly publicized on television, radio, and the internet. There is no enforcement or evaluation conducted on these strategies and community participation is implicit in their success. And finally, the MoH conducted reactive outdoor insecticide fogging in the vicinity of reported cases. Reactive fogging was designed to treat areas 100m around houses with a clinically diagnosed dengue case, approximately 1 week post-diagnosis.

These vector interventions, however, did not significantly reduce dengue incidence in Makassar, based upon data beginning in 1999. In this report, we describe how the addition of a targeted, dry season insecticide application, in addition to the standard MoH procedures, is associated with a reduction and continued low dengue incidence in Makassar for 8 years in a programmatic, non-research context.

## Methods

### Site description

The study area was metropolitan Makassar City (5.14°S, 119.43°E; elevation < 25 m), the capital of South Sulawesi Province. Greater Makassar covers more than 2,500 sq. km and had a population in 2017 of approximately 1.4 million (Data from National Center of Statistics Agency). Administratively the city is divided into 143 villages and about 1,000 sub-villages. South Sulawesi is subject to the Indo-Australian monsoon (rain) cycle, with heavy rains occurring during November-April followed by a relatively dry season, May-October. Rainfall data were provided by the Indonesian Agency for Meteorology, Climatology, and Geophysics (*BMKG*).

### Dengue case surveillance

The MoH has routinely collected and tabulated clinically suspected and laboratory confirmed dengue fever and severe dengue cases based on World Health Organization (WHO) criteria [30] since 1999. Cases documented were based on MoH official monthly reports for the city of Makassar and other cities in South Sulawesi. The national procedure for reporting dengue (clinically suspected, diagnosed as dengue, and with a platelet count less then 100,000/ul) is followed in all districts) with no differences in reporting methodologies over the course of the observational period.

### Vector surveillance

Beginning in September 2006, at the expected end of the dry season, *Aedes* immature surveys were conducted by 2 local volunteers in and around houses in every sub-village, focusing on artificial containers containing water. The MoH supervised surveillance. With homeowner consent, each pair of surveyors would inspect 50–100 randomly selected houses in each sub-village (each sub village had 100 to 400 houses). A household was approached, and if consent was given, the house was surveyed; if not, the volunteers continued to neighboring houses. Volunteers trained in identifying *Aedes* mosquitoes counted all larvae and pupae in containers having diameters <2m, with no minimum size. Data was first tabulated at sub-district primary health clinics and then submitted to the MoH at Makassar for analysis and evaluation before being communicated to Universitas Hasanuddin (UNHAS). When feasible, habitats containing Aedes larvae, or with the potential to harbor them, were emptied. *Aedes*-positive sub-villages were identified on maps and the number of houses positive for immature *Aedes* were used to calculate a house index (HI): the number of houses with positive *Aedes* immature divided by the number of houses surveyed, expressed as a percentage. Pre-season Fogging: The threshold for the additional village fogging in 2006 and 2007, was set at HI > 40%, that is, *Aedes* immatures discovered in >40% of houses surveyed. This threshold was decreased to 35% from 2008 to 2010, and then further decreased to 25% after 2011. Since the observed temporal drop in HI would result in fewer areas being part of the pre-season fogging, the HI threshold was decreased to maximize available funding and capacity for fogging. The HI data for 2006 and 2007 could not be obtained from the MoH, and pre-season fogging was not conducted in 2013 and 2014. Villages with a threshold HI as described above were fogged, using backpack apparatus, with 5% Malathion in diesel oil. Fogging by trained MoH personnel took place once, over the entire village, about 4 weeks after the larval survey analyses were completed. A 4–6 member team, each supplied with one fogger, was able to treat an entire village area. Since the local population did not usually permit indoor fogging, the fogging was usually performed outdoors during the day, targeting the airspace in the peri-domestic area.

### Study procedure overview

Larval surveys–characterized by searching the entire premise for water containers harboring immature mosquitoes–both inside and outside houses, with a focus on man-made containers less than 2m in diameter, were performed between September and October (the peak of the dry season), and pre-emptive mass fogging was usually implemented from late September to late October for 2–4 weeks. At the end of each transmission season the HI data were compared with reported dengue and rainfall data to evaluate the impact of spraying. Adult entomological indices were not compiled. Routine MoH vector control interventions—including larval source management (LSM), larvaciding, and regular MoH reactive fogging (fogging in a 100m diameter radius around houses of reported dengue cases), were not interrupted. The frequency and density of MoH reactive fogging (also called focal fogging) was based on the number of cases reported. The pre-emptive, pre-season fogging was conducted, monitored and evaluated from 2006 to 2012; monitoring for associated reductions in cases was stopped in 2014.

### Analysis

Since this data is likely Poisson-distributed, a non-parametric test (Mann Whitney U test) was performed to compare the annual incidence of reported dengue recorded by the MOH Makassar before (2003–2006) and after (2007–2014) the pre-emptive fogging approach was implemented. A comparison of reported dengue incidence reduction in Makassar City relative to the overall cases reported in South Sulawesi, the cases from cities flanking Makassar City (Gowa and Maros), and the other main cities in South Sulawesi (with the Makassar cluster excluded) was performed. This presentation of these observations may be considered an ‘interrupted time series’ [31], with data present before and after the intervention in the absence of a control [32]—though comparisons were made to unmatched non-intervention cities as well as neighboring cities.

## Results

**Fig 1:**
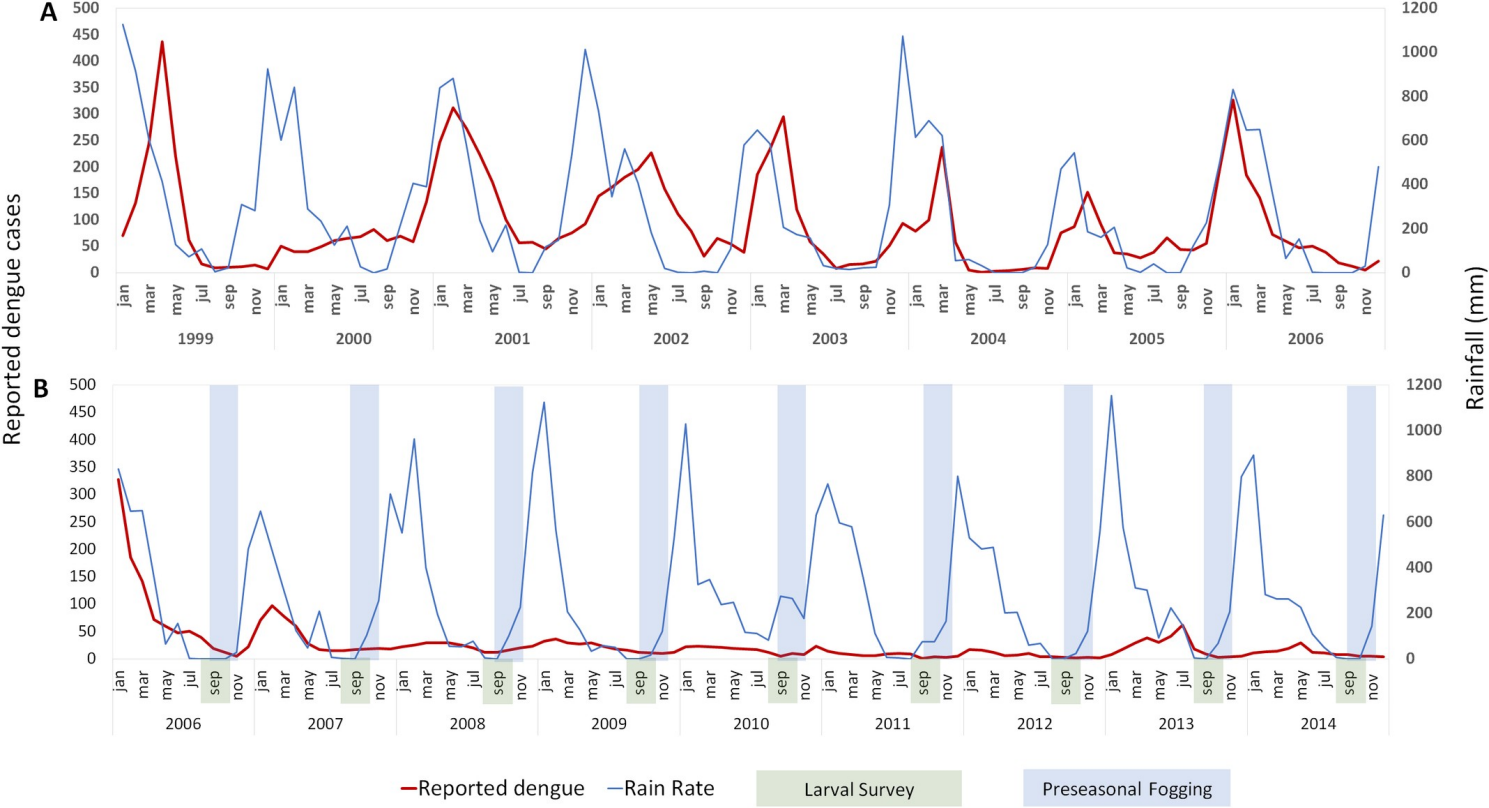
Relationship between rainfall and cases of Dengue as reported by the Ministry of Health (MoH) of Makassar City. (**A**) A slight lag seen in increased Dengue cases and the onset of rainfall during the transmission season every year before the intervention. **(B)** After implementation of the revised program of larval surveys followed by fogging in selected areas based on HI, an immediate and sustained decrease is seen in dengue cases for 8 years. Green boxes denote the period of larval surveys, while the blue boxes depict the period of the additional pre-emptive, dry-season fogging, Note that rainfall does not significantly change pre and post intervention.

The addition of a targeted, dry season insecticide application, in addition to the standard MoH procedures, is associated with a reduction of dengue incidence. Clinical reports of dengue and rainfall are compared for 1999–2014 in Fig 1. The period 1999–2005 preceded the dry-season intervention (Fig 1A), which was implemented during 2006–2012 (Fig 1B). Rainfall patterns remained seasonal and consistent with historical norms for the 8-year period. The additional pre-season fogging in 2006 was followed by a reduction of dengue cases during the subsequent rainy season (Fig 1B). Based on this result, pre-seasonal surveys and interventions continued in 2007 with surveys and fogging at the end of the dry season (Fig 1B), in addition to routine MoH activities.

**Fig 2:**
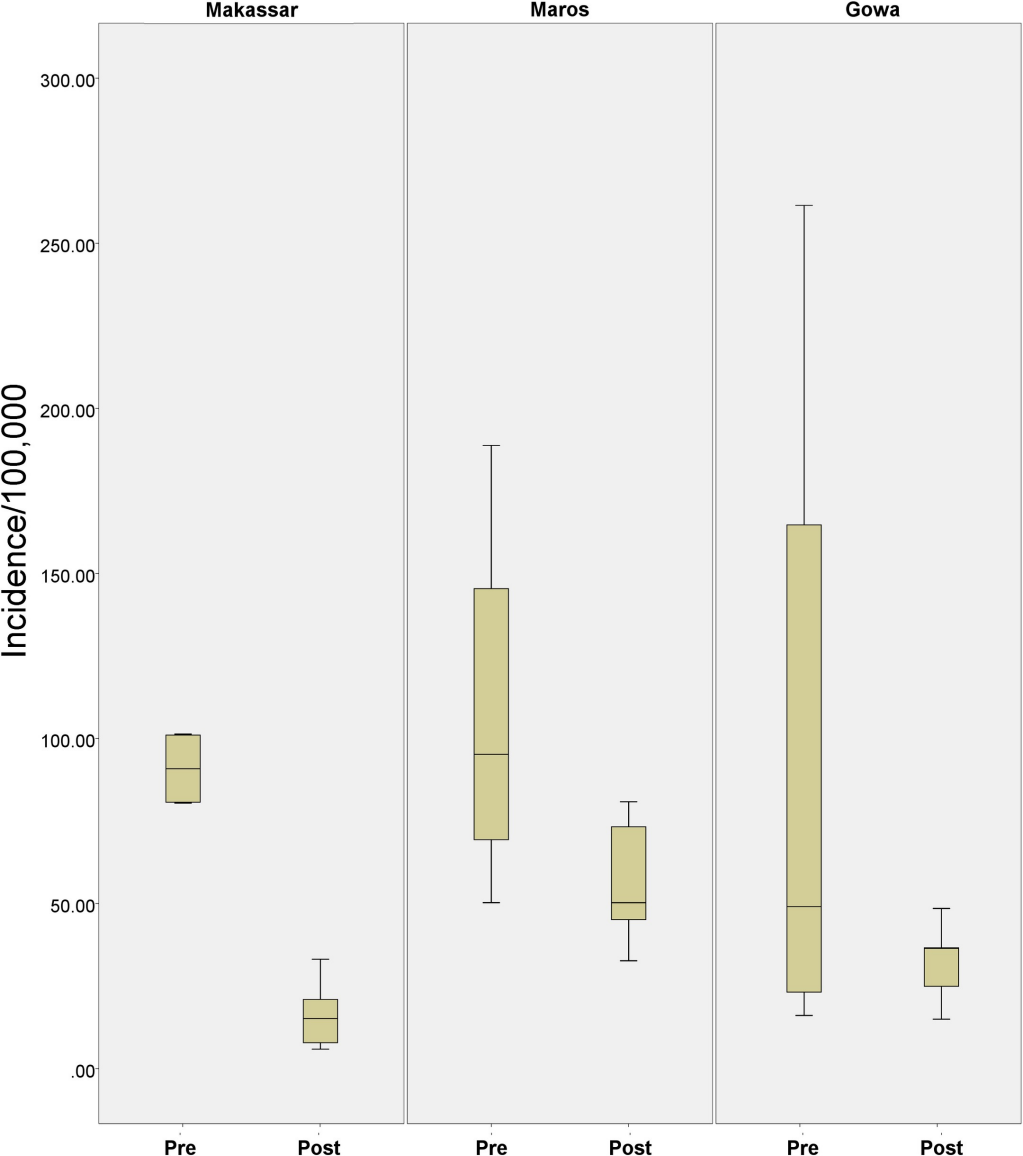
A Box-plot demonstrating the differences in incidence in Makassar and the neighboring cities of Maros and Gowa, pre (2003–2006) and post (2007–2014) intervention. There was a significant reduction in incidence in Makassar pre and post intervention (Mann Whitney U test, p = 0.007), a decrease in Maros (p = 0.042), and a non-significant decrease in Gowa (p = 0.497). Population level data (and hence incidence calculations) were only present from 2003 onwards.

Reported dengue cases sharply declined following the implementation of late dry season, targeted fogging. From 2003–2006, annual reported incidence ranged from 81.1 to 101.4 cases per 100,000. In the 2007 transmission season, following the first intervention, incidence dropped to 33.3 cases per 100,000), compared to 938 the previous year. In the following years (2007–2014) incidence further dropped and ranged from 6.0 to 21.4 cases per 100,000. The statistically significant (Mann Whitney U test; p < 0.007, Fig 2 and S1 Text) decline suggests that the reduction of cases after the addition of pre-emptive interventions was not random.

**Fig 3:**
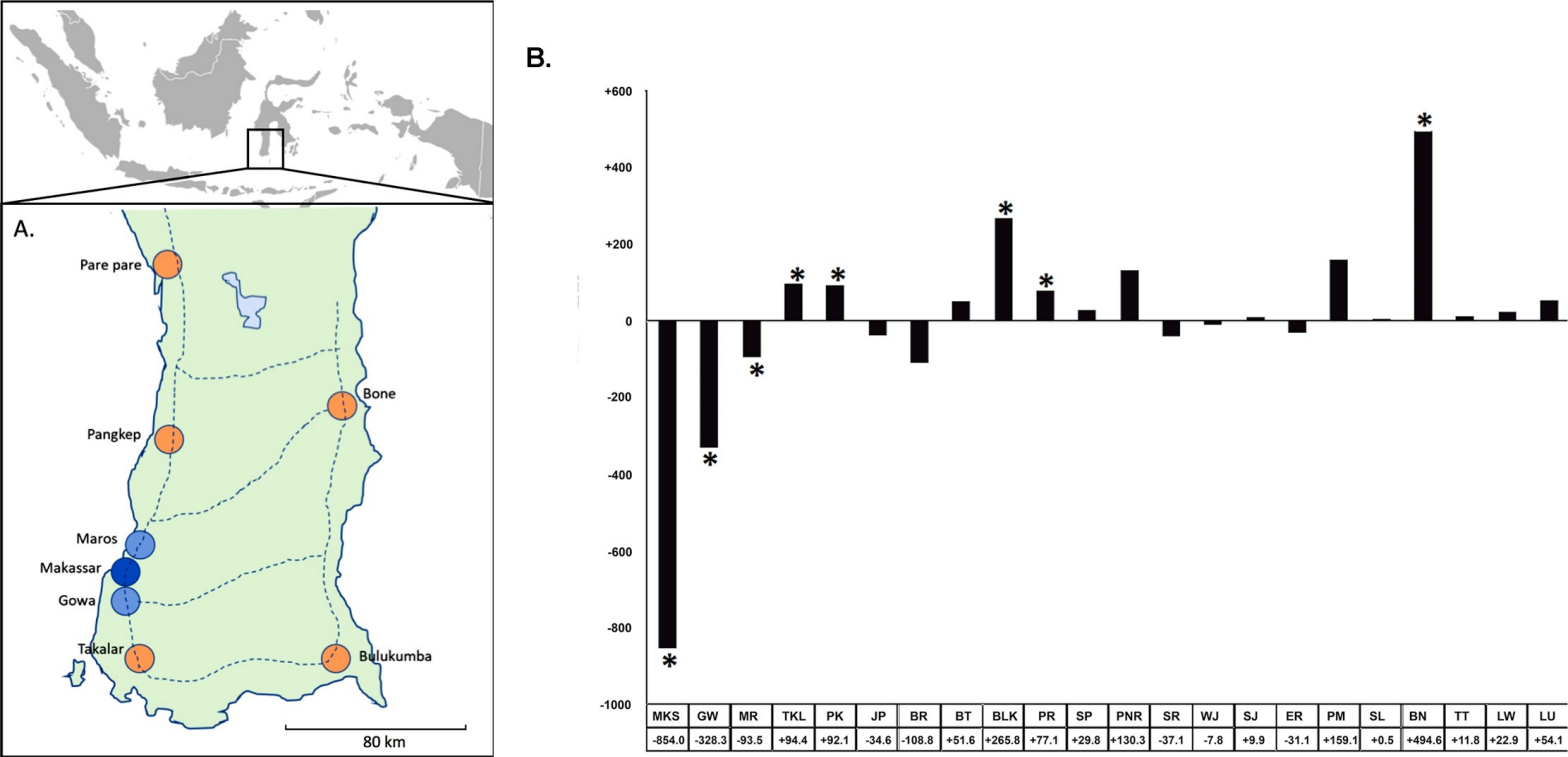
**A) Distance visualization of several cities been compared to Makassar on its dengue annual cases.** The cities of Gowa and Maros that flank Makassar are depicted along with the location of the five largest cities outside Makassar. The inset depicts the location of South Sulawesi within Indonesia. Blue indicates cities with decreased reported dengue while orange indicates cities with increased dengue (Map Source: Hand drawn). **B) Change in annual reported cases post intervention in South Sulawesi.** Change in average annual reported dengue cases post intervention in the 23 major cities in South Sulawesi in order of distance away from Makassar. An * denotes cities on the map in (A). MKS = Makassar*, GW = Gowa*, MR = Maros*, TKL = Takalar*, PK = Pangkep*, JP = Jeneponto, BR = Barru, BT = Bantaeng, BLK = Bulukumba*, PR = Pare-Pare*, SP = Soppeng, PNR = Pinrang, SR = Sidrap, WJ = Wajo, SJ = Sinjai, ER = Enrekang, PM = Polmas, SL = Selayar, BN = Bone*, TT = Tana Toraja, LW = Luwu, LU = Luwu Utara.

As reported cases decreased in Makassar city, dengue cases increased elsewhere in South Sulawesi Province (Fig 3). Makassar’s share of all reported dengue cases in South Sulawesi was 34.5% for the pre-intervention period 2003 to 2006, but only 6.9% in 2007 to 2014. Post-intervention reported dengue decreased by 80.4% in Makassar, compared with an increase of 222% in 19 of the 21 next largest cities in South Sulawesi. Incidence also fell in 2 cities contiguous with Makassar but where no intervention was performed. Though not as significant as in Makassar, reported cases in Gowa and Maros fell during the monitoring period 62% and 35%, respectively, from their levels before the intervention was started in Makassar (Fig 3). Though not as significant as was seen in Makassar, incidence dropped in both Maros and Gowa. Incidence in Gowa (2003–2005), ranged from 30.2 to 261.5 cases per 100,000, while in Maros it ranged from 50.3 to 188.8 cases per 100,000. From 2006 to 2014 incidence dropped to a low of 15 cases per 100,000 in Gowa (range of 15 to 48.5 cases per 100,000) and in Maros to 32.7 cases per 100,000 (a range of 32.7 to 102.0 cases per 100,000).

**Fig 4:**
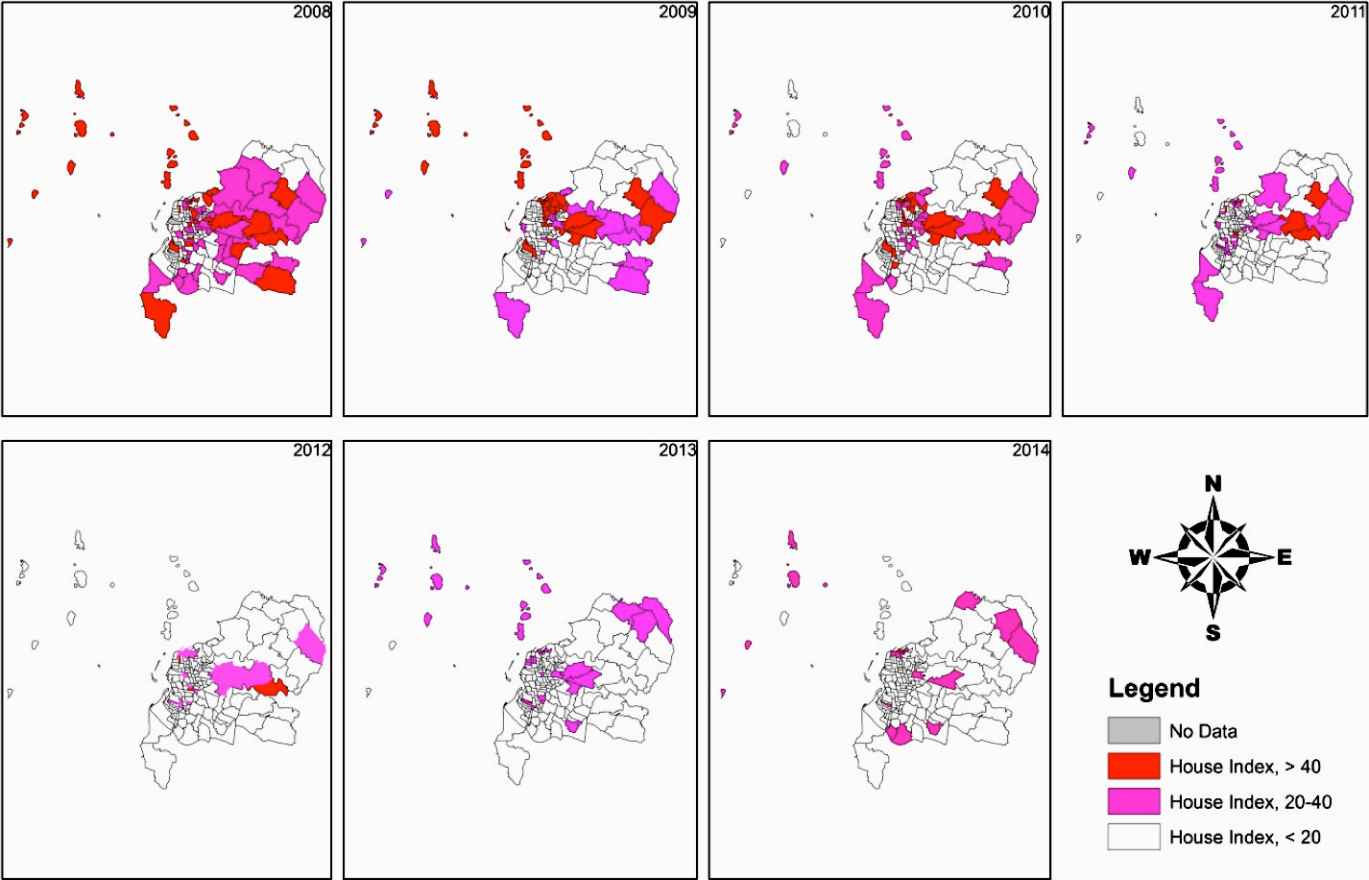
Reduction of *Aedes* Larval Densities—House Indexes (HI), in Makassar from 2008 to 2014. HI is indicated by color: Red = greater than 40%, Orange = 35% to 40%, Pink = 25% to 34% and white = 0% to 24%. (Map Source: ArcGIS 10.6).

During the larval surveys, > 90% of mosquito immatures found in containers near or in houses in Makassar were *Ae*. *aegypti* or *Ae*. *albopictus* (S4 Text). A decrease in HI was observed across the entire Makassar city from 2008 onwards (Fig 4 and S2 Text). Twenty-five villages with HI > 40 were sprayed before the rainy season in 2008. This number decreased over time, and only 1 village had an HI > 40 in 2014 (Fig 4) demonstrating both a reduced density and spatial distribution of *Aedes* immatures.

## Discussion

This retrospective analysis of operational data demonstrates that a single annual application of adulticide, timed for the end of the dry season, was accompanied by a profound reduction in both larval indices as well as reported symptomatic dengue cases—when added to the existing regimen of reactive fogging around the houses diagnosed cases. The rationale for initiating this procedure in 2006 was the hypothesis that by waiting to begin Aedes interventions until the first cases were reported, the first wave of transmission initiated by the beginning of the rains, was already underway. Because many asymptomatic dengue infections are capable of infecting mosquitoes [33], by delaying control measures until cases appeared risked the buildup of a sizable, covert, infective population. The lag between the onset of rains and the first recorded cases was 4–6 weeks.

Makassar is representative of many rapidly urbanizing tropical cities in supporting an abundance of mosquito oviposition sites, especially for *Ae*. *aegypti*. It is also representative in being at high dengue risk. Its seasonal rainfall schedule can be predicted with considerable certainty, making the described protocol feasible. As seen in the data for 1999–2006, cycles of rainfall, *Aedes* density, and human dengue cases were correlated.

The data we analyzed were generated from a change in operational control procedure, which was not designed as a research study, but may be considered a programmatic evaluation using an interrupted time series approach. Consequently, our conclusions are tempered by several limitations. The most important is that there were no matched experimental control areas where all procedures were known to be identical except for pre-seasonal fogging. During the eight years examined dengue cases increased in all metropolitan areas of South Sulawesi, with two exceptions, while dramatically decreasing in the augmented vector suppression area of Makassar. The two exceptions, Gowa and Maros, are both contiguous with the augmented suppression area. We cannot exclude the possibility that some unrecognized factor or factors independent of pre-season fogging and exclusive to only Makassar, Gowa, and Maros was responsible for much or all the decrease in reported dengue cases in all three. Because case reporting throughout South Sulawesi is under the supervision of the provincial health department, it is unlikely that there was a prolonged lapse in clinical surveillance, which would have reduced reporting in only Gowa and Maros. We observed that the very limited routine vector surveillance was maintained, or increased as part of this implementation, in Makassar during the observational period, while standard MoH epidemiological surveillance and reporting remained the same. It is important to note that the boundaries between the three contiguous cities (Makassar, Gowa and Maros) are administrative, and not geographical barriers. The observed reduction in vectors in Makassar in combination with the reduction in the virus reservoir would have extended into the areas spatially closest (Maros and Gowa) to Makassar—fits with the “source and sink” mechanism [34, 35]. Understanding the effects of vector control interventions on non-target and adjacent geographic areas should be an important part of an intervention strategy–to both vector control managers and scientists.

There were no demographic changes–a possible confounder, with respect to the compositions of the population. The primary change was an increase of the total population (from about 1.15 million to 1.41 million, National Statistics Agency, Indonesia), which did not result in a corresponding proportional increase in dengue.

Although there were no presumptive treatments in 2013 and 2014, cases remained low. In both years, however, the reactive vector suppression treatments in the vicinity of cases continued. Because data more recent than 2014 were not available for analysis, we do not know if cases have gradually increased to levels representative of elsewhere in South Sulawesi. There were also gaps in methodology and inconsistencies in data collection. Methods for intervention did not always remain the same during the eight years; the HI threshold for pre-rainy season fogging became increasingly stringent, decreasing from 40% to 35% to 25%—these changes were based on maximizing available finances and capacity (for fogging) in combination with the reduced number of areas meeting the HI-fogging threshold. The large number of volunteers suggest that in any given year there might have been lack of uniformity in the data on which HI were calculated, however, the large number of intervention units would have reduced the effect of systematic error. No data were collected on adult *Aedes* densities. Reliance on immature indices is typical in most dengue suppression programs but a more direct correspondence between adult vectors and humans would have provided additional insight. Here, the HI (and its consequent spatial and temporal reduction) may be considered reflective of the spatial distribution of blood feeding and egg-laying female Aedes that oviposit around houses. Routine insecticide susceptibility testing would also have been valuable; limited resistance tests performed using CDC bottle assays on local *Ae*. *aegypti* in 2004 and 2006 demonstrated no Malathion resistance (S3 Text). Though insecticide resistance was not observed, the emergence and spread of resistance is an important factor in any control strategy. This can be mitigated by rotating insecticides used or using them in mosaic patterns [36]. Volunteers were trained to identify *Aedes* larvae but it was not logistically feasible for the MoH to subsequently confirm them as *Ae*. *aegypti*, *Ae*. *albopictus*, or other Stegomyia. Previous data demonstrated that nearly all *Aedes* larvae in the types of containers sampled in the area were either *Ae*. *aegypti* or *Ae*. *albopictus* (S4 Text).

There is a consensus that maximum impact in reducing vector populations may be achieved when control interventions are implemented with high coverage and are integrated with other control strategies [19, 37]. A single thermal fogging application will not result in long lasting protection and will have a low probability of affecting intradomiciliary populations of *Aedes* [18, 21]. This study associates a reduction in dengue incidence with a single pre-emptive chemical intervention in *addition* to good case surveillance and targeted interventions in the vicinity of cases.

Ultimately, successful control must be suited to local transmission dynamics. The strategy we describe here has the advantage of only modifying existing procedures by altering the timing and locations of fogging. While the costs for increased surveys and fogging might be greater, they are almost certainly outweighed by the savings in reduced dengue incidence [38, 39]. Since *Ae*. *aegypti* is also the primary vector of chikungunya and Zika viruses, which are endemic in Indonesia, the prevention of epidemics caused by those pathogens cannot be discounted.

A vital measure of this report, including all its limitations, is that it is based on the normal function, capability, and capacity of a MoH team implementing a community intervention. These remarkable results warrant more robust, comprehensive studies designed to test the preemptive intervention hypothesis.

## Supporting information

S1 Text The Mann Whitney U test, was used to calculate significant differences between independent groups: a) Pre and post implementation of the intervention in Makassar. b) Pre and post implementation between Makassar, Maros and Gowa–neighboring cities.
(DOCX)

S2 Text House Index (HI) changes over time for each village in Makassar. Red cells: HI above 40%, Orange cells: HI above 20%, below 40%, Yellow cells: HI below 20%.
(DOCX)

S3 Text Insecticide susceptibility tests of Aedes aegypti to Malathion in a) 2004 (CDC bottle assays) and in b) 2006 (larval susceptibility test).
(DOCX)

S4 Text The number of larvae (*Aedes* and *Culex*) found in containers in villages in Makassar (2008). (DOCX)
