# Supplementary material for: Integrated vector management with additional pre-transmission season thermal fogging is associated with a reduction in dengue incidence in Makassar, Indonesia: Results of an 8-year observational study
Source: PLoS Negl Trop Dis. 2019 Aug 5;13(8):e0007606. doi: 10.1371/journal.pntd.0007606 (PMC6695203; doi:10.1371/journal.pntd.0007606)
Supplement: S1 Text — The Mann Whitney U test, was used to calculate significant differences between independent groups: a) Pre and post implementation of the intervention in Makassar. b) Pre and post implementation between Makassar, Maros and Gowa–neighboring cities. (DOCX) [file pntd.0007606.s001.docx]

**S1 Text.** The Mann Whitney U test, was used to calculate significant differences between independent groups

**a. Makassar:**

| **Ranks** | | | | |
| --- | --- | --- | --- | --- |
|  | Period | N | Mean Rank | Sum of Ranks |
| Incidence | Pre | 4 | 10.50 | 42.00 |
|  | Post | 8 | 4.50 | 36.00 |
|  | Total | 12 |  |  |

| **Test Statistics^a^** | |
| --- | --- |
|  | Incidence |
| Mann-Whitney U | .000 |
| Wilcoxon W | 36.000 |
| Z | -2.717 |
| Asymp. Sig. (2-tailed) | .007 |
| Exact Sig. [2*(1-tailed Sig.)] | .004^b^ |
| a. Grouping Variable: period | |
| b. Not corrected for ties. | |

**b. Neighboring cities:**

| **Ranks** | | | | | |
| --- | --- | --- | --- | --- | --- |
| Neighbour cities | | period | N | Mean Rank | Sum of Ranks |
| Gowa | incidence | Pre | 4 | 7.50 | 30.00 |
|  |  | Post | 8 | 6.00 | 48.00 |
|  |  | Total | 12 |  |  |
| Maros | incidence | Pre | 4 | 9.50 | 38.00 |
|  |  | Post | 8 | 5.00 | 40.00 |
|  |  | Total | 12 |  |  |
| Makassar | incidence | Pre | 4 | 10.50 | 42.00 |
|  |  | Post | 8 | 4.50 | 36.00 |
|  |  | Total | 12 |  |  |

| **Neighboring cities** | | **incidence** |
| --- | --- | --- |
| Gowa | Mann-Whitney U | 12.000 |
|  | Wilcoxon W | 48.000 |
|  | Z | -.679 |
|  | Asymp. Sig. (2-tailed) | .497 |
|  | Exact Sig. [2*(1-tailed Sig.)] | .570^b^ |
| Maros | Mann-Whitney U | 4.000 |
|  | Wilcoxon W | 40.000 |
|  | Z | -2.038 |
|  | Asymp. Sig. (2-tailed) | .042 |
|  | Exact Sig. [2*(1-tailed Sig.)] | .048^b^ |
| Makassar | Mann-Whitney U | .000 |
|  | Wilcoxon W | 36.000 |
|  | Z | -2.717 |
|  | Asymp. Sig. (2-tailed) | .007 |
|  | Exact Sig. [2*(1-tailed Sig.)] | .004^b^ |
| a. Grouping Variable: period | | |
| b. Not corrected for ties. | | |
