# Supplementary material for: Integrated vector management with additional pre-transmission season thermal fogging is associated with a reduction in dengue incidence in Makassar, Indonesia: Results of an 8-year observational study
Source: PLoS Negl Trop Dis. 2019 Aug 5;13(8):e0007606. doi: 10.1371/journal.pntd.0007606 (PMC6695203; doi:10.1371/journal.pntd.0007606)
Supplement: S2 Text — Red cells: HI above 40%, Orange cells: HI above 20%, below 40%, Yellow cells: HI below 20%. (DOCX) [file pntd.0007606.s002.docx]

**S2 Text.** House Index (HI) changes over time for each village in Makassar_._  Red cells: HI above 40%, Orange cells: HI above 20%, below 40%, Yellow cells: HI below 20%

_
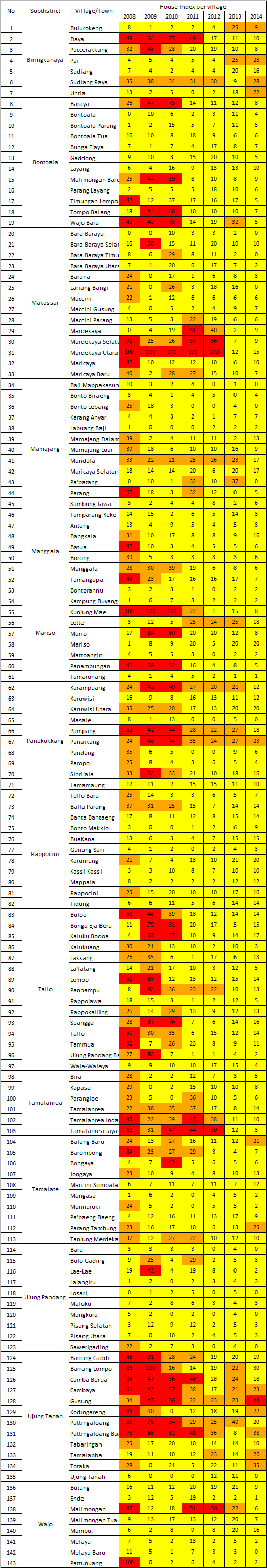
_**­­­­­­**
