# Supplementary material for: Integrated vector management with additional pre-transmission season thermal fogging is associated with a reduction in dengue incidence in Makassar, Indonesia: Results of an 8-year observational study
Source: PLoS Negl Trop Dis. 2019 Aug 5;13(8):e0007606. doi: 10.1371/journal.pntd.0007606 (PMC6695203; doi:10.1371/journal.pntd.0007606)
Supplement: S3 Text — Insecticide susceptibility tests of Aedes aegypti to Malathion in a) 2004 (CDC bottle assays) and in b) 2006 (larval susceptibility test). (DOCX) [file pntd.0007606.s003.docx]

**S3 Text.** Insecticide susceptibility tests of *Aedes aegypti* to Malathion in a) 2004 (CDC bottle assays) and in b) 2006 (larval susceptibility test).

**a. Year 2004**

| **Site Malathion use in the last 10 years** | **% Mortality with Malathion 0.04%** | | | | |  |
| --- | --- | --- | --- | --- | --- | --- |
|  | **0 minutes** | **5 minutes** | **10 minutes** | **15 minutes** | **1 hour** | **Test results** |
| 0 (never) | 0 | 9.9 | 88.1 | 100 | 100 | susceptible |
| 1-3 (less frequent) | 0 | 16.2 | 86.8 | 100 | 100 | susceptible |
| 4-7 (frequent) | 0 | 12.5 | 89 | 97.6 | 100 | susceptible |
|  |  |  |  |  |  |  |
| **Site Malathion use in the last 10 years** | **% Mortality with Malathion 1%** | | | | |  |
|  | **0 minutes** | **5 minutes** | **10 minutes** | **15 minutes** | **1 hour** | **Test results** |
| 0 (never) | 0 | 73.8 | 100 | 100 | 100 | susceptible |
| 1-3 (less frequent) | 0 | 59.6 | 93.7 | 100 | 100 | susceptible |
| 4-7 (frequent) | 0 | 48.7 | 93.5 | 100 | 100 | susceptible |
|  |  |  |  |  |  |  |
| **Site Malathion use in the last 10 years** | **% Mortality with Malathion 5%** | | | | |  |
|  | **0 minutes** | **5 minutes** | **10 minutes** | **15 minutes** | **1 hour** | **Test results** |
| 0 (never) | 0 | 100 | 100 | 100 | 100 | susceptible |
| 1-3 (less frequent) | 0 | 71.3 | 100 | 100 | 100 | susceptible |
| 4-7 (frequent) | 0 | 65.1 | 98.8 | 100 | 100 | susceptible |

**b. Year 2006**

| **Site with malathion exposure in the last 10 years** | **% Aedes Larva Mortality with Malathion 0.00084 %** | | | | | |
| --- | --- | --- | --- | --- | --- | --- |
|  | **0 minutes** | **30 minutes** | **60 minutes** | **90 minutes** | **120 minutes** | **Test results** |
| 0 (never) | 0 | 23.1 | 100 | 100 | 100 | susceptible |
| 1-3 (less frequent) | 0 | 9.2 | 73.5 | 100 | 100 | susceptible |
| 4-7 (frequent) | 0 | 8.4 | 53.9 | 80.3 | 89.0 | Resistance possible |
