# Supplementary material for: Integrated vector management with additional pre-transmission season thermal fogging is associated with a reduction in dengue incidence in Makassar, Indonesia: Results of an 8-year observational study
Source: PLoS Negl Trop Dis. 2019 Aug 5;13(8):e0007606. doi: 10.1371/journal.pntd.0007606 (PMC6695203; doi:10.1371/journal.pntd.0007606)
Supplement: S4 Text — (DOCX) [file pntd.0007606.s004.docx]

**S4 Text.** The number of larvae of *Aedes* (either *Ae. aegypti* or *Ae. albopictus*) and *Culex* spp. found in containers in villages in Makassar (2008).

| **No** | **Subdistrict** | **Village** | **# Containers checked** | **# Containers positive** | **# *Aedes* positive** | **# *Culex* positive** | **% Aedes** | **% Culex** |
| --- | --- | --- | --- | --- | --- | --- | --- | --- |
| 1 | Biringkanaya | Daya | 359 | 177 | 175 | 3 | 98.9 | 1.7 |
| 2 | Biringkanaya | Pai | 160 | 20 | 20 | 0 | 100 | 0 |
| 3 | Bontoala | Bontoala Tua | 218 | 34 | 33 | 1 | 97.1 | 2.9 |
| 4 | Bontoala | Wajo Baru | 251 | 54 | 54 | 0 | 100 | 0 |
| 5 | Makassar | Mardekaya Selatan | 202 | 142 | 137 | 5 | 96.5 | 3.5 |
| 6 | Makassar | Maricaya Baru | 260 | 103 | 101 | 2 | 98.1 | 1.9 |
| 7 | Mamajang | Mamajang Dalam | 180 | 70 | 70 | 0 | 100 | 0 |
| 8 | Mamajang | Mamajan g Luar | 212 | 71 | 70 | 1 | 98.6 | 1.4 |
| 9 | Manggala | Borong | 380 | 147 | 145 | 2 | 98.6 | 1.4 |
| 10 | Manggala | Tamangapa | 340 | 149 | 149 | 1 | 100 | 0.7 |
| 11 | Mariso | Mattoangin | 160 | 6 | 6 | 0 | 100 | 0 |
| 12 | Mariso | Tamarunang | 200 | 33 | 33 | 0 | 100 | 0 |
| 13 | Panakukkang | Pampang | 100 | 51 | 50 | 1 | 98 | 2 |
| 14 | Panakukkang | Paropo | 200 | 134 | 132 | 2 | 98.5 | 1.5 |
| 15 | Panakukkang | Tello Baru | 274 | 67 | 65 | 3 | 97 | 4.5 |
| 16 | Tallo | Lakkang | 80 | 25 | 25 | 0 | 100 | 0 |
| 17 | Tallo | Pannampu | 120 | 9 | 9 | 0 | 100 | 0 |
| 18 | Tallo | Ujung Pandang Baru | 100 | 27 | 27 | 0 | 100 | 0 |
| 19 | Tamalanrea | Kapasa | 240 | 66 | 66 | 0 | 100 | 0 |
| 20 | Tamalanrea | Parangloe | 220 | 50 | 50 | 0 | 100 | 0 |
| 21 | Tamalanrea | Tamalanrea Indah | 309 | 140 | 138 | 2 | 98.6 | 1.4 |
| 22 | Tamalate | Balang baru | 310 | 3 | 3 | 0 | 100 | 0 |
| 23 | Tamalate | Barombong | 470 | 396 | 392 | 5 | 99 | 1.3 |
| 24 | Tamalate | Tanjung Merdeka | 240 | 89 | 86 | 3 | 96.6 | 3.4 |
| 25 | Ujung Padang | Pisang Selatan | 160 | 4 | 4 | 0 | 100 | 0 |
| 26 | Ujung Padang | Pisang Utara | 280 | 19 | 19 | 0 | 100 | 0 |
| 27 | Ujung Tanah | Tabaringam | 200 | 117 | 115 | 2 | 98.3 | 1.7 |
| 28 | Ujung Tanah | Ujung Tanah | 140 | 102 | 102 | 0 | 100 | 0 |
| 29 | Wajo | Butung | 120 | 19 | 19 | 0 | 100 | 0 |
| 30 | Wajo | Ende | 158 | 5 | 5 | 0 | 100 | 0 |
